# Models of multiple system atrophy

**DOI:** 10.1038/s12276-019-0346-8

**Published:** 2019-11-18

**Authors:** He-Jin Lee, Diadem Ricarte, Darlene Ortiz, Seung-Jae Lee

**Affiliations:** 1 Department of Anatomy, School of Medicine, Konkuk University, 120 Neungdong-Ro, Gwangjin-gu, Seoul, 05029 South Korea; 2 Research Institute of Medical Science, Konkuk University, Seoul, 05029 South Korea; 3 IBST, Konkuk University, Seoul, 05029 South Korea; 4 Department of Medicine and Biomedical Sciences, Seoul National University College of Medicine, Seoul, 03080 South Korea

**Keywords:** Parkinson's disease, Neurodegeneration

## Abstract

Multiple system atrophy (MSA) is a neurodegenerative disease with diverse clinical manifestations, including parkinsonism, cerebellar syndrome, and autonomic failure. Pathologically, MSA is characterized by glial cytoplasmic inclusions in oligodendrocytes, which contain fibrillary forms of α-synuclein. MSA is categorized as one of the α-synucleinopathy, and α-synuclein aggregation is thought to be the culprit of the disease pathogenesis. Studies on MSA pathogenesis are scarce relative to studies on the pathogenesis of other synucleinopathies, such as Parkinson’s disease and dementia with Lewy bodies. However, recent developments in cellular and animal models of MSA, especially α-synuclein transgenic models, have driven advancements in research on this disease. Here, we review the currently available models of MSA, which include toxicant-induced animal models, α-synuclein-overexpressing cellular models, and mouse models that express α-synuclein specifically in oligodendrocytes through cell type-specific promoters. We will also discuss the results of studies in recently developed transmission mouse models, into which MSA brain extracts were intracerebrally injected. By reviewing the findings obtained from these model systems, we will discuss what we have learned about the disease and describe the strengths and limitations of the models, thereby ultimately providing direction for the design of better models and future research.

## Neurodegenerative disease: how to study multiple system atrophy

A review of the models available for studying multiple system atrophy (MSA), a Parkinson’s-like disease, may help identify new treatment options. MSA is difficult to diagnose and unresponsive to drugs. Similar to Parkinson’s disease, it involves accumulation of protein aggregates in brain and spinal cord cells, but the causes are poorly understood. He-Jin Lee at Konkuk University, and Seung-Jae Lee at Seoul National University College of Medicine in South Korea and coworkers have reviewed the models available to study the disease, including toxin-induced and transgenic animal models, and recent evidence that transferring the protein aggregates into cells causes MSA symptoms. Each model mimics some aspects of the disease, but none captures the full range of symptoms. This review helps highlight research pathways that may illuminate treatments for this complex and debilitating adult-onset disease.

## Introduction

Multiple system atrophy (MSA) is a rapidly progressive sporadic adult-onset neurodegenerative disorder. It was first termed to describe neuronal atrophy found in various diseases, including striatonigral degeneration, olivopontocerebellar atrophy, and Shy-Drager syndrome^1^. Epidemiologic studies of MSA have shown a prevalence range of 3.4 to 4.9 per 100,000 people, increasing to 7.8 per 100,000 among people older than 40 years of age^2^. Moreover, it affects men and women equally and has an average age onset of approximately 55–60 years^3,4^. The mean life expectancy of MSA is 6–10 years following diagnosis^5,6^.

MSA is characterized by clinical symptoms that are subdivided into extrapyramidal, pyramidal, cerebellar, and autonomic symptoms. Extrapyramidal symptoms include bradykinesia, rigidity, and postural instability, which are similar to the symptoms of Parkinson’s disease (PD). The autonomic symptoms include common autonomic dysfunctions, such as urogenital, gastrointestinal, and cardiovascular failure. Nonmotor symptoms, such as sleep and cognitive disorders, respiratory problems, and emotional/behavioral symptoms, might also occur during disease development^7,8^. The different symptoms of MSA can be used to categorize the disease into two subtypes: the parkinsonian subtype (MSA-P) and the cerebellar type (MSA-C). MSΑ−P patients exhibit more PD symptoms, whereas MSA-C patients commonly display cerebellar ataxia. More than two-thirds of MSA patients in Western countries are MSA-P patients, while MSA-C is more common in Japan^9–11^ However, MSA-P is more common than MSA-C in Korea, indicating that the subtypes of MSA vary among Asian countries^12,13^.

**Fig. 1:**
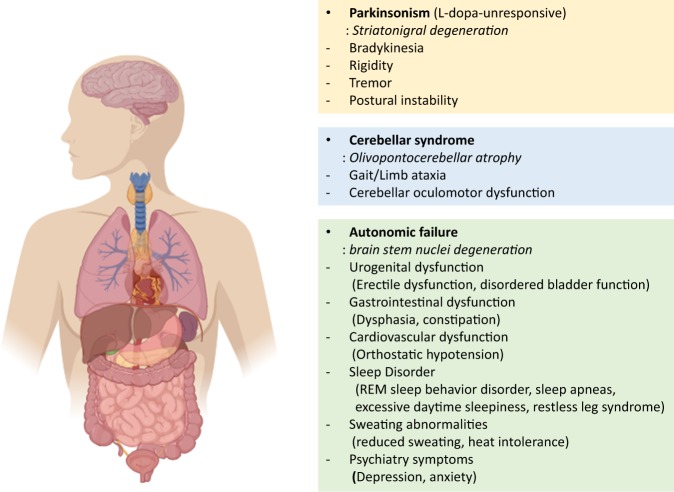
Clinical characteristics of MSA. The symptoms are subdivided into parkinsonian, cerebellar, and autonomic. Parkinsonian symptoms include motor symptoms, such as bradykinesia, tremor, and postural instability. Patients with more evident parkinsonism are considered to have the parkinsonian subtype of the disease (MSA-P). Patients with cerebellar symptoms, such as ataxia and cerebellar oculomotor dysfunction, are considered to have the cerebellar subtype of the disease (MSA-C). Both subtypes share common autonomic dysfunctions described above.

MSA is pathologically distinguished by a widespread neuronal loss that is accompanied by gliosis in the basal ganglia, cerebellum, pons, inferior olivary nuclei, and spinal cord. The symptoms of MSA are similar to those of PD, which makes it difficult to distinguish the two diseases. Clinical differential diagnosis is practically possible, although neuropathological confirmation is still required for a definitive diagnosis of MSA (Fig. 1).

## Multiple system atrophy and glial cytoplasmic inclusions

The important neuropathological hallmark of MSA is the presence of argyrophilic filamentous glial cytoplasmic inclusions (GCIs), predominantly in oligodendrocytes^14^. GCIs are spherical protein aggregates located near nuclei with a diameter of 5–20 μm and various morphologies. GCIs in oligodendrocytes are usually larger and paler than nonoligodendrocyte-derived GCIs. They are primarily composed of loosely packed filaments of α-synuclein protein that is phosphorylated at residue Ser129 and ubiquitinated^15,16^. Immunohistochemical studies have identified other proteins that colocalize with α-synuclein. These include p25α/TPPP (tubulin polymerization-promoting protein), α,β-crystallin, tau, LRRK2, cyclin-dependent kinase 5 (cdk5), microtubule-associated protein 5, ubiquitin, and tubulin (reviewed in ref. ^17^). p25α/TPPP has a vital role in the stabilization of microtubules, the projection of mature oligodendrocytes, and ciliary structures^18^. It is essential for the differentiation and maturation of oligodendrocytes^19^. p25α/TPPP is commonly found in myelin sheaths, but during the first stages of MSA, it relocates to the oligodendrocyte soma, resulting in early myelin dysfunction^20^. The redistribution of p25α in oligodendrocytes causes an increase in the volume of cell bodies, which is a typical characteristic of cells with GCIs. Ultimately, the presence of p25α in the cell body enhances the aggregation of α-synuclein, which may lead to oligodendroglial dysfunction and neuronal degeneration^18,21^.

## Multiple system atrophy and α-synuclein

MSA belongs to a diverse group of neurodegenerative disorders described as α-synucleinopathies, which are similar to PD and dementia with Lewy bodies (DLB). These disorders are characterized by the abnormal accumulation of α-synuclein protein aggregates^22,23^. α-Synuclein is a predominantly neuronal presynaptic protein present in the brain and is expressed in other tissues at various levels. It is encoded by the SNCA gene, which is linked to PD and has also been associated with an increased risk of PD, DLB, and MSA^24^.

The presence of GCIs and the excessive accumulation of α-synuclein in the oligodendrocytes are accompanied by neuronal degeneration, brain atrophy, demyelination, and mutation of nerve cells in MSA patients^25,26^. A study by Peng et al. showed differences between GCI-α-synuclein and LB-α-synuclein^27^. These inclusions are conformationally and biologically distinct. GCI-α-synuclein is 1000-fold more potent than LB-α-synuclein in seeding the aggregation of monomeric α-synuclein, which may explain the highly aggressive and rapidly progressive nature of MSA symptoms.

Several studies have reported that there is little to no α-synuclein expression in mature oligodendrocytes in the human brain^11,28,29^. There is no evidence of increased α-synuclein expression in MSA oligodendrocytes. However, a recent study by Asi et al. showed a three-fold increase in SNCA mRNA levels in MSA oligodendrocytes postmortem, although the change did not reach statistical significance; it is still questionable whether the increase in mRNA levels can significantly change the levels of the α-synuclein protein in oligodendrocytes^30^. In vitro cultures of MSA patient-derived induced pluripotent stem cells (iPSCs) showed that α-synuclein is only expressed in the early stages of oligodendrocyte maturation but not in the premyelination period^31^.

The mechanisms of the accumulation of α-synuclein in oligodendrocytes are still unknown. Several hypotheses have provided possible explanations as to how GCIs form^32^. One possibility is that they form through the induced expression and aggregation of α-synuclein in oligodendrocytes and other glial cells under disease conditions, but there is little evidence to support this cell-autonomous mechanism. An alternative explanation is that they form through the uptake of α-synuclein secreted from neurons by oligodendrocytes. Studies have shown the transfer of neuronal α-synuclein, both in co-cultures and through exogenous addition, into oligodendrocytes in vitro and in vivo^33–35^. In MSA, oligodendrocytes might be more prone than neuronal cells to the accumulation of neuron-derived α-synuclein, possibly because the clearance mechanism might not be as efficient as that in neurons. In support of this explanation, Peng et al. suggested that the cellular milieu determines different synucleinopathies, such as LBD and MSA^27^.

**Fig. 2:**
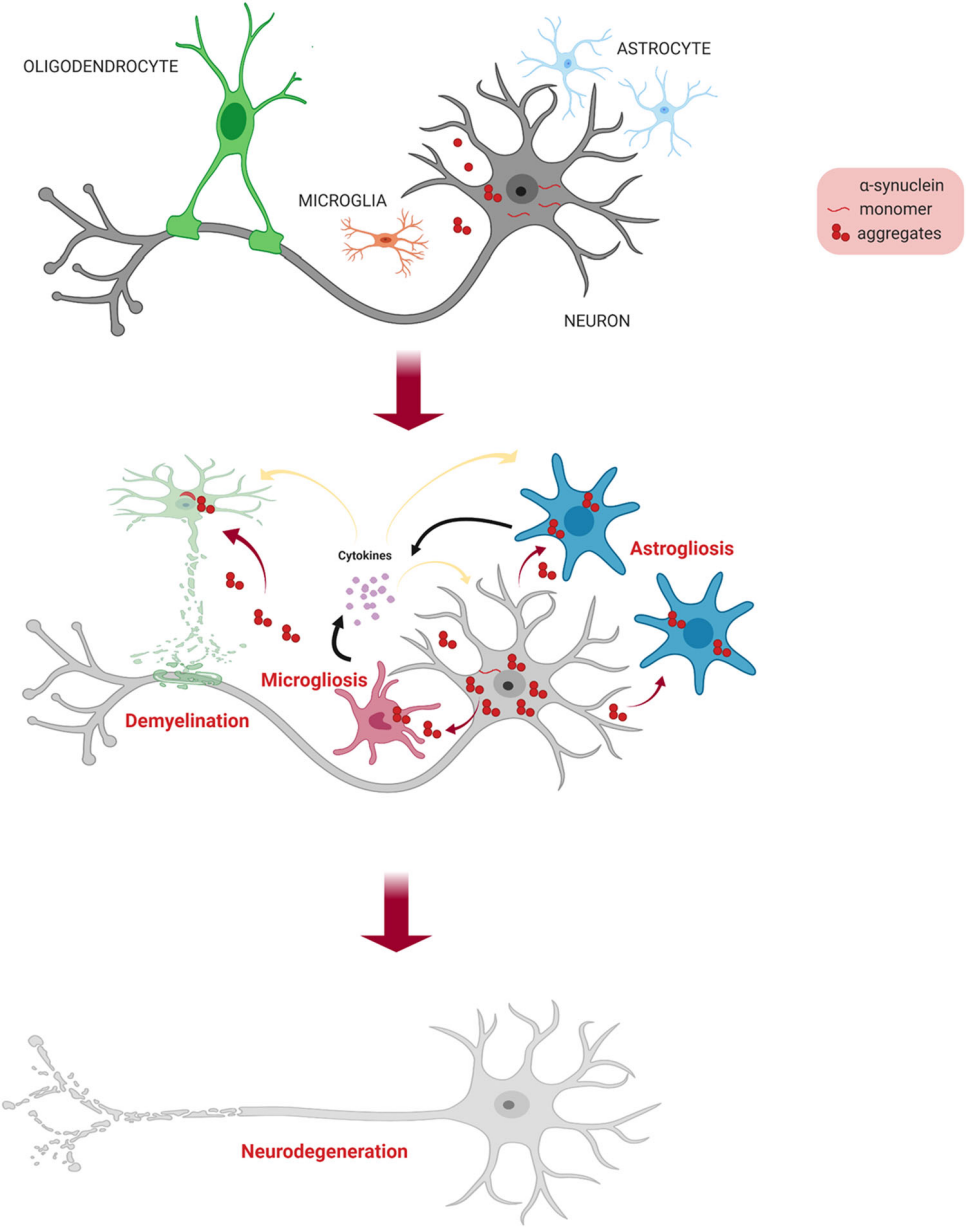
The transmission model of α-synuclein in the brain. Neuron-derived α-synuclein aggregates in the extracellular space are taken up by neighboring glial cells. Microglia and astrocytes, which secrete inflammatory molecules, are activated. Oligodendrocytes undergo demyelination, exposing neuronal axons that may retract or be degenerated by the hostile external environment, causing neurodegeneration.

A failure to discard proteins through cellular degradation pathways may lead to the production of toxic aggregates that may incorporate into GCIs and cause oligodendrocyte dysfunction. Oligodendrocytes become enlarged, and nuclei turn pale when myelin degeneration occurs. The propagation of α-synuclein aggregates to other adjacent cells may lead to inflammatory responses by microglia and neurodegeneration (Fig. 2).

## Astrogliosis and microgliosis in MSA

The activation of astrocytes and microglia has been observed in the brains of MSA patients, as well as in those of transgenic models of MSA^11,36,37^. Studies have revealed the potential role of the neuron-to-glia transmission of α-synuclein in glial activation in both cell and animal models. Extracellular α-synuclein leads to inflammatory responses in astrocytes and microglia^38–40^. Activated Iba-1-positive microglia and GFAP-positive astrocytes have been shown to localize in the proximity of GCIs^41,42^. Astrogliosis is an important pathological characteristic of MSA. Treating astrocytes with extracellular α-synuclein induces ERK/MAPKK-dependent astrogliosis^42^. Activated astrocytes can secrete cytokines, which may trigger microgliosis. Therefore, the proinflammatory function of extracellular α-synuclein in astrocytes may have a crucial role in spreading MSA neuropathology^41,43^.

Microglia are the primary immunophagocytic cells in the brain. An increased number of activated microglia is found in α-synucleinopathies^44^. The injection of GCI extract into the mouse brain causes localized microgliosis, as well as astrogliosis^42^. Toll-like receptors (TLRs), such as TLR2 and TLR4, have been shown to interact with extracellular α-synuclein in microglia^39,45^. Microglia activated by extracellular α-synuclein then secrete toxic factors that can trigger further neurodegeneration and gliosis^46^.

## Models of multiple system atrophy

### Animal toxin models

In vitro and in vivo models have been developed to obtain a better understanding of MSA pathophysiology. The systemic administration and local stereotaxic injection of toxins induced lesions in specific anatomical areas of models to reproduce MSA symptoms, particularly L-DOPA-unresponsive parkinsonism^47^. The stereotaxic injection of two toxins, namely, 6-hydroxydopamine (6-OHDA) and quinolinic acid (QA), into different regions of the rat brain in sequence had distinct pathological and behavioral outcomes^48^. 6-OHDA caused striatal dopamine depletion and decreased the number of dopaminergic cells in the substantia nigra pars compacta (SNpc)^49^. The injection of QA induced the loss of spiny striatal neurons. Behavioral deficits induced by both single and combined lesions also imitated the symptoms of MSA-P. However, there was no significant difference in performance on the rotarod test or drug-induced rotation test^50^. The inoculation of brain regions with a single toxin also created lesions. The injection of 3-nitropropionic acid (3-NP), a succinate dehydrogenase inhibitor, or 1-methyl-4-phenylpyridinium ion (MPP^+^), a mitochondrial complex I inhibitor, into the striatum induced combined degeneration of nigral and striatal neurons^51,52^. The simultaneous or sequential systemic administration of 1-methyl-4-phenyl-1,2,3,6-tetrahydropyridine (MPTP) and 3-NP caused motor symptoms and dyskinesia in mice and nonhuman primates that responded poorly to L-DOPA treatment^53–56^.

These approaches partially mimicked MSA neuropathology in the nigrostriatal system, which arises in the early to late period of the disease. The lesions only resulted in symptoms within the area of administration and failed to spread outside the basal ganglia^47^. In addition, toxin-based approaches did not induce GCI pathology, which is one of the essential hallmarks of MSA^47^.

### In vitro genetic models

In vivo toxin models cannot precisely depict disease advancement or the mechanism. MSA pathophysiology has been addressed at the cellular level through modified gene expression. Because one of the main components of GCIs in MSA is α-synuclein, many studies have used in vitro expression of α-synuclein to investigate the disease mechanism^32^.

#### U-373 MG cell line and primary mixed rat glial cultures

A study conducted by Stefanova et al. showed that the overexpression of α-synuclein induced cell death in a U-373 MG human glioblastoma astrocytoma cell line and primary oligodendrocytes from mixed rat glial cultures^32,57^. Glial cells expressing high levels of α-synuclein were highly prone to oxidative stress. Upon treatment with TNFα, a proinflammatory cytokine released by microglia in MSA, significant cytotoxic changes were observed in α-synuclein-expressing cells. This suggested that a toxic environment, along with high levels of α-synuclein in glia, might represent a severe risk for the development of MSA.

#### OLN-93 cell line

OLN-93 cells, primary oligodendrocytic cells derived from Wistar rat brain cultures, have been used to study MSA pathology^32,58^. Kragh and colleagues co-expressed α-synuclein and p25α in the OLN-93 cell line. The co-expression resulted in the enhanced expression of IkBα, which sequestered the NF-κB transcription factor p65 in the cytoplasm. The inhibition of NF-κB signaling impeded its cytoprotective effects while causing the retraction of microtubules and triggering the activation of the apoptotic protein caspase-3^58,59^. The phosphorylation of Ser129 in α-synuclein protein was necessary for the process. Human brain tissues from MSA patients also exhibit increased expression of IkBα and NFκB p65 in some oligodendrocytes with GCIs.

#### CG4 cell line

The central glia 4 (CG4) cell line, a rodent oligodendroglial cell line, has been used to stably express α-synuclein. The overexpression of the protein impaired the maturation of cells into oligodendrocytes. BDNF partially rescued this impairment^32,60^. α-Synuclein delayed the maturation of oligodendroglia by inhibiting the expression of myelin basic protein (MBP) and myelin gene regulatory factor while increasing the levels of the oligodendroglial differentiation repressor Hes5. The lentiviral transduction of α-synuclein in CG4 cells impaired autophagy and this impairment was reversed by the inhibition of miR-101, a microRNA that regulates autophagy genes such as RAB5A, MTOR, ATG4D, and STMN1^61^.

#### iPSCs from human MSA patients

The advancement of stem cell technologies has allowed the culturing of patient-derived cells and their differentiation into various cell types, including oligodendrocytes. The expression of α-synuclein was examined by real-time PCR and immunocytochemistry. Oligodendrocyte precursor cells (OPCs) expressed α-synuclein, but the expression levels were decreased as the cells differentiated into mature oligodendrocytes. There was no difference between patient-derived and healthy control iPSCs^31^.

### In vivo genetic models

The transgenic expression of the α-synuclein gene under oligodendrocyte-specific promoters has been used to create mouse models of MSA. Through the use of proteolipid protein (PLP), MBP, and the cyclic nucleotide 3’-phosphodiesterase (CNP) promoters, α-synuclein was exclusively expressed in oligodendrocytes^62–64^. However, transgenic mouse lines overexpressing α-synuclein displayed different pathological features of MSA under different oligodendroglial promoters. In these models, motor and nonmotor symptoms developed to varying degrees. Furthermore, these models exhibited accumulation of α-synuclein and its aggregates in oligodendrocytes similar to GCIs in MSA patients.

In addition, adeno-associated viruses (AAVs) were genetically modified to express α-synuclein, specifically in oligodendrocytes. Using these recombinant viruses, more diverse animal models, such as nonhuman primates, can be established^65,66^. However, the various symptoms described in each of these models and the pattern and degree of degeneration in the nervous system did not precisely correlate with disease pathology^47,67^.

#### PLP-hαSyn transgenic mice

PLP promoter-driven α-synuclein (PLP-hαSyn) transgenic mice bred on a C57/BL6 background exhibited phosphorylation of α-synuclein at Ser129 and aggregation of α-synuclein along with GCI-like inclusions^62^. Mitochondrial inhibition by 3-NP in these mice induced the degeneration of the striatonigral system and gliosis^68^. The loss of dopaminergic neurons in the SNpc, Purkinje cells, and neurons in the pons and medulla oblongata, as in the human MSA-C subtype, was also observed. Treatment with 3-NP augmented motor deficits in tg mice compared to non-tg controls at ~12 months. Autonomic symptoms, however, appeared earlier, as early as 2 months of age^69^. Urinary bladder dysfunction with morphological changes in the bladder wall and increased postvoid residual volumes was detected at 2 months of age^70^. Heart rate variability, an indication of changed sympathovagal balance, as observed in the human disease, was reduced at 5 months of age^71^. At the age of 13 months, respiratory failure was observed^72^. Progressive motor deficits emerged at 6 months and progressed until 18 months of follow-up. The loss of dopaminergic neurons in the SNpc was found during the initial stages. The loss of striatal dopaminergic terminals and DARPP32-positive projection neurons was observed at 12 months^73^. Gliosis and increased levels of cytokines were also reported.

#### MBP-hαSyn transgenic mice

Mice with MBP promoter-driven α-synuclein expression in oligodendrocytes exhibited somewhat different pathological features of MSA that those exhibited by PLP-hαsyn and CNP-hαsyn tg mice^63^. These mice displayed demyelination along with axonal degeneration in the cerebellum, basal ganglia, brain stem, corpus callosum, and neocortex. The high expressor line (line 29) died prematurely at 6 months. The moderate expressor line (line 1) exhibited a mild phenotype with disease onset after 6 months of age. α-Synuclein accumulation was also evident, along with elevated astrogliosis. 3-NP administration increased nitrated and oxidized α-synuclein levels but not the levels of the phosphorylated or total protein^74^. Neurological deficits were augmented and accompanied by widespread neurodegeneration and behavioral problems. Increases in neuroinflammation were detected in regions of high α-synuclein expression, such as the corpus callosum and the striatum, even before symptoms were evident^75^. Inflammatory responses were restricted to myeloid cells, and severe astrogliosis was only detected in gray matter regions. RNA sequencing of α-synuclein-expressing primary oligodendrocytes demonstrated upregulation of cytokines and genes important for inflammatory responses, suggesting that neuroinflammation may be critical for disease development. MBP-hαsyn tg mice displayed GDNF deficiency, IκBα and miR-96 upregulation, and a delay in oligodendrocyte maturation^59,69,76,77^.

#### CNP-hαSyn transgenic mice

CNP promoter-hαSyn mice exhibited the accumulation of fibrillary human α-synuclein in oligodendrocytes, a loss and demyelination of these cells, and severe gliosis in the brain and spinal cord^64,69^. The aggregation of endogenous mouse α-synuclein, which is associated with neuronal loss, was also detected in the axons and axon terminals of spinal cord neurons. Motor deficits started at the age of 7 to 9 months, but the phenotypes seem to be different from typical MSA symptoms^78–80^.

In general, the accumulation of α-synuclein in oligodendroglia has been found in all three oligodendrocyte-specific promoter-driven human α-synuclein tg mice. However, each of these mice displayed distinct patterns of pathological changes that do not precisely replicate human MSA pathology^47,81^.

#### Viral-mediated oligodendroglial α-synuclein expression models

Bassil and colleagues utilized AAVs to express human α-synuclein under the control of the MBP promoter in adult Sprague-Dawley (SD) rats and nonhuman primates (macaques)^65^. α-Synuclein expression was observed in ~80% of oligodendrocytes in the injected area of SD rats. The injected rats displayed L-DOPA-unresponsive motor deficits by 6 months of age, while significant dopaminergic cell loss occurred at 3 months of age. Increased amounts of the insoluble and phosphorylated forms of human α-synuclein were detected in the striatum and SNpc of injected rats. The injected macaques showed expression of human α-synuclein in ~60% of oligodendrocytes and ~40% of neurons. It has yet to be determined whether the neuronal α-synuclein came from the direct expression of α-synuclein in neurons or from the oligodendrocyte-to-neuronal transmission of α-synuclein. Longitudinal studies are also required to determine if viral-mediated oligodendroglial α-synuclein expression is useful as an MSA model.

A study by Mandel et al. utilized the oligotrophic AAV vector Olig001, which specifically transduces oligodendrocytes, to express human α-synuclein in nonhuman primates^66^. The injection of the recombinant vector into the striatum of rhesus macaques induced widespread expression of α-synuclein in more than 90% of oligodendrocytes throughout the striatum by 3 months of age. Phosphorylated α-synuclein was detected, and the GCI-like inclusions in oligodendrocytes were resistant to proteinase K (PK) digestion. Demyelination in the white matter tracts of the corpus callosum and microgliosis in the striatum was also detected.

The viral expression of α-synuclein in animals has advantages over genetic models in that expression can be manipulated temporally. The recombinant virus can be injected at an early stage or later in development. The cell specificity of viral infection has significantly improved over the years. However, more studies of such models in rodents and nonhuman primates are still needed for the development of MSA models.

### Transmission models

Numerous studies have reported little to no α-synuclein expression in mature oligodendrocytes in vitro and in vivo^11^. The mechanisms of the accumulation of α-synuclein aggregates in the oligodendrocytes and astrocytes of MSA patients are still largely unknown. However, recent reports of the transfer of α-synuclein from one cell to another, particularly from neurons to glia, have led to the hypothesis that toxic α-synuclein aggregates secreted from neurons are taken up by oligodendrocytes and disrupts their function^35,38^. Based on this hypothesis, several groups have developed transmission models.

#### In vitro culture models of exogenously added α-synuclein aggregates

Pukas et al. utilized primary rat brain oligodendrocytes and an oligodendroglial cell line, OLN-93, and added soluble or preaggregated forms of human recombinant α-synuclein^82^. Both forms of exogenously added α-synuclein were internalized and formed small intracellular aggregates in oligodendroglial cells. Under oxidative stress, the levels of aggregates increased, and cell viability was reduced. Human α-synuclein preformed fibrils (PFFs) triggered the expression and aggregation of endogenous α-synuclein in rat primary OPCs^83^. However, when using PFFs, one should consider that the PFFs may differ from neuron-derived α-synuclein aggregates in structure and composition. In addition, there may be essential cofactors secreted from surrounding cells that influence the transmission of the protein.

In vitro co-culture models of α-synuclein-expressing donor cells and naïve recipient cells have been established. Neuron-to-neuron, neuron-to-astrocyte, and neuron-to-microglia transmission of α-synuclein have recently been reported^38,39,84^. The transfer of α-synuclein to recipient cells caused various cellular changes, including cell toxicity and inflammatory responses. The advantage of using such models is that it is easy to manipulate the expression of specific genes and to detect cellular changes by biochemical methods

The use of co-cultures of neurons and oligodendrocytes was reported recently. Mouse embryonic stem cell (ESC)-derived neural progenitor cells (NPCs) differentiated into OPCs and then into mature oligodendrocytes. These cells were co-cultured with differentiated cortical neurons from mouse ESCs on the same plate. However, unlike in other studies in which neurons overexpressed α-synuclein and were co-cultured with other naïve recipient cells, this study utilized direct lentiviral overexpression of α-synuclein in oligodendrocytes, which induced myelination deficits upon co-culture with naïve neurons^85^. Neuron-to-oligodendrocyte transmission and its effects remain to be studied.

#### Stereotaxic injection of recombinant proteins or MSA brain extracts into animal models

Brain extracts from 14 MSA patients were inoculated into the brains of TgM83+/− hemizygous mice expressing the A53T α-synuclein mutant under the prion promoter^86^. All mice showed neurodegeneration and accumulation of α-synuclein aggregates in neuronal cell bodies and axons at 4 months postinjection, whereas control injections did not induce neurodegeneration at the same age. Interestingly, PD brain extracts did not promote the aggregation of α-synuclein in TgM83 mice, indicating that pathogenic α-synuclein species may differ in MSA and PD. The same group performed comparison experiments with different transgenic animal models expressing wild-type, A30P, and A53T α-synuclein on a mouse α-synuclein knockout background^87^.

In contrast to the results obtained in TgM83+/− mice, these mice, when injected with MSA brain extracts, did not show any motor deficits, even after 330–400 days. Only A53T-expressing (Tg(SNCA*A53T^+/+^)) mice, but not others, displayed α-synuclein pathology in neurons and astrocytes in the limbic system. Interestingly, these injected mice retained infectivity after the initial inoculation of human MSA patient samples. Mouse brain extracts from MSA patient extract-injected tg mice were inoculated for the second time into both TgM83+/− and Tg(SNCA*A53T^+/+^) mice. These second-round synucleinopathy models also developed pathology, and the transmission of α-synuclein was confirmed.

These injection models support the α-synuclein transmission hypothesis, as pathological α-synuclein from diseased patients was transferred to nonsymptomatic mice and induced pathology.

## Conclusion

**Fig. 3:**
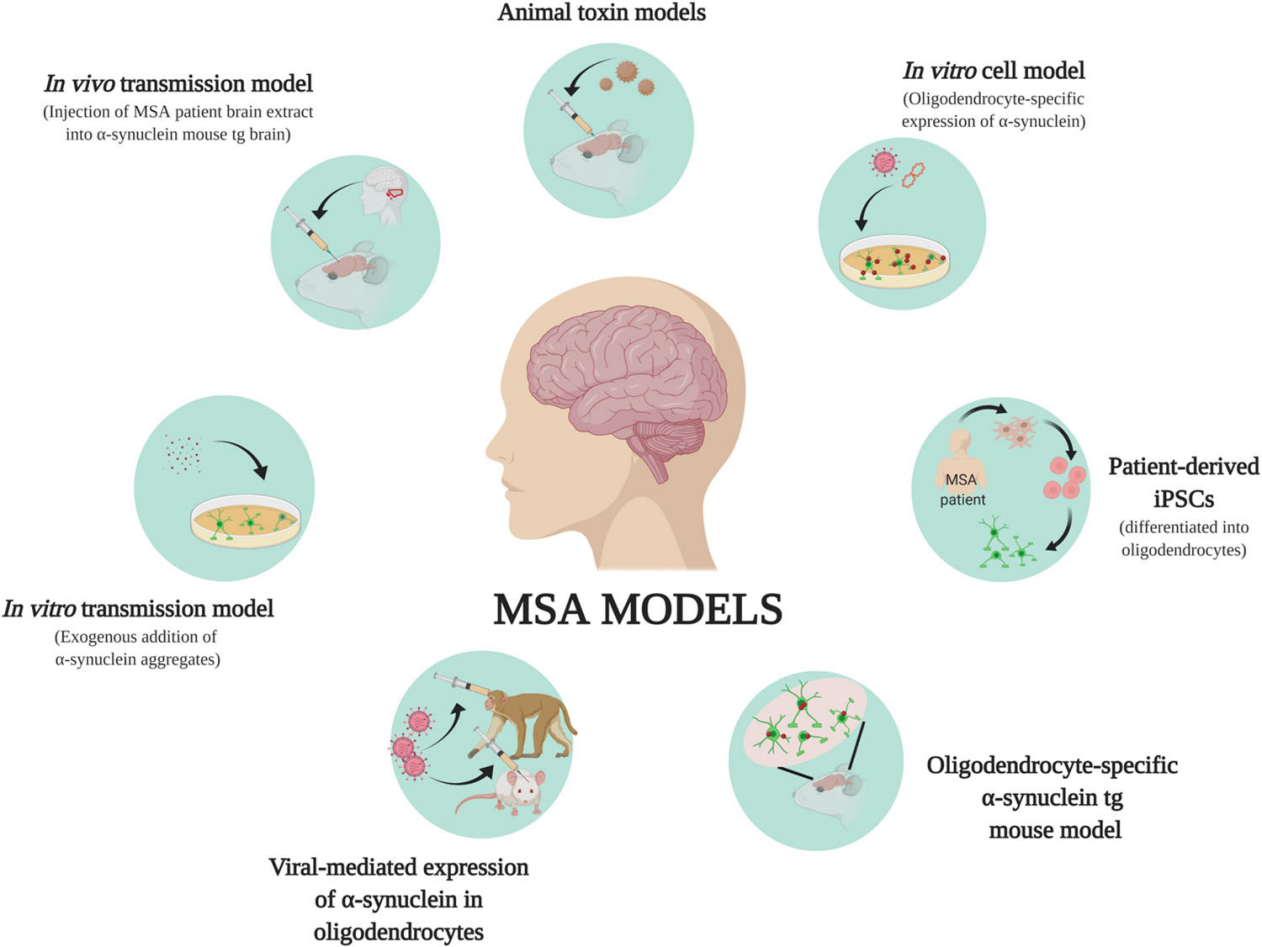
Summary of MSA models. In vitro cell models have been used to express α-synuclein in oligodendrocytes. The effects of the addition of exogenous α-synuclein aggregates to oligodendrocytes have been studied in in vitro transmission models. Recent developments in stem cell technologies have allowed the growth of MSA-derived iPSCs and their differentiation into oligodendrocytes. Comparison studies of RNA, protein, and epigenetic changes in normal and patient-derived cells are ongoing. Animal models induced by the injection of toxins exhibit MSA-like symptoms, and oligodendrocyte-specific expression of α-synuclein has been achieved through the generation of tg mice and viral-mediated expression.

MSA is a neurodegenerative disease with clinical symptoms similar to those of PD and cerebellar ataxia. The complexity and rapid progression of the disease, as well as its unresponsiveness to drugs, such as L-DOPA for parkinsonian symptoms, make MSA a challenging disease to treat. Various models have been generated over the years to study the mechanism of MSA development and progression. In this review, we summarized toxin-induced models, in vitro and in vivo genetic models, and transmission models for studying α-synuclein pathology and behavioral symptoms in MSA (Fig. 3).

The main goal of the genetic models is to generate oligodendrocytes with GCI-like α-synuclein pathology, thereby allowing us to study the roles of α-synuclein in MSA pathogenesis. Two methods have been applied to generate models with oligodendrocytic α-synuclein accumulation. The first method was to ectopically express α-synuclein in oligodendrocytes. α-Synuclein was overexpressed in oligodendroglial cells in culture. Transgenic animal models expressing α-synuclein specifically in oligodendrocytes were created. These direct expression models displayed glial α-synuclein pathology and various CNS phenotypes akin to those of MSA. However, because mature oligodendrocytes have little to no α-synuclein expression, it is not clear whether these ectopic expression models are valid representatives of human MSA.

More recent efforts to develop MSA models have utilized the fact that α-synuclein can be transferred between cells. The exogenous addition of recombinant α-synuclein aggregates to oligodendrocytes resulted in the uptake and induction of endogenous protein aggregate formation. The stereotaxic injection of human MSA brain extracts into the brains of α-synuclein tg mice induced extensive spreading of α-synuclein pathology, neuroinflammation, and neurodegeneration. However, these injected mouse models displayed neuronal α-synuclein pathology instead of oligodendroglial pathology.

Although researchers have been able to recapitulate some aspects of MSA with current models, none of these models present bona fide MSA pathology, which is represented by oligodendroglial GCIs generated from endogenous α-synuclein proteins. Recent studies have suggested that GCIs are generated through the transfer of α-synuclein from neurons to oligodendrocytes. To test this hypothesis, we will have to understand more about the biology of oligodendrocytes in MSA. Critical questions include how oligodendrocytes respond to and process neuron-derived α-synuclein and how normal oligodendrocytes and MSA oligodendrocytes respond differently to α-synuclein. We anticipate that the technical advancement of single-cell analysis and bioinformatics will contribute to answering these questions.
